# Bacterial Composition and Diversity of the Digestive Tract of *Odontomachus monticola* Emery and *Ectomomyrmex javanus* Mayr

**DOI:** 10.3390/insects12020176

**Published:** 2021-02-17

**Authors:** Zhou Zheng, Xin Hu, Yang Xu, Cong Wei, Hong He

**Affiliations:** 1 College of Forestry, Northwest A&F University, Yangling 712100, Shaanxi, China; zhouzheng@nwafu.edu.cn (Z.Z.); Huux15@163.com (X.H.); yangxu19880511@gmail.com (Y.X.); 2 Key Laboratory of Plant Protection Resources and Pest Management, Ministry of Education, College of Plant Protection, Northwest A&F University, Yangling 712100, Shaanxi, China

**Keywords:** bacterial communities, ponerine ants, guts, infrabuccal pockets, social insects, high-throughput sequencing

## Abstract

**Simple Summary:**

Bacteria are considered to be one of the compelling participants in ant dietary differentiation. The digestive tract of ants is characterized by a developed crop, an elaborate proventriculus, and an infrabuccal pocket, which is a special filtrating structure in the mouthparts, adapting to their special trophallaxis behavior. Ponerine ants are true predators and a primitive ant group; notably, their gut bacterial communities get less attention than herbivorous ants. In this study, we investigated the composition and diversity of bacterial communities in the digestive tract and the infrabuccal pockets of two widely distributed ponerine species (*Odontomachus monticola* Emery and *Ectomomyrmex javanus* Mayr) in northwestern China using high-throughput sequencing of the bacterial 16S rRNA gene. The results revealed that, not only do the gut bacterial communities display significant interspecies differences, but they also possess apparent intercolony characteristics. Within each colony, the bacterial communities were highly similar between each gut section (crops, midguts, and hindguts) of workers, but significantly different from their infrabuccal pockets, which were similar to bacterial communities in larvae of *O. monticola*. The relationship of the bacterial communities among the infrabuccal pockets, gut sections and larvae provide meaningful information to understand the social life and feeding behavior of ants.

**Abstract:**

Ponerine ants are generalist predators feeding on a variety of small arthropods, annelids, and isopods; however, knowledge of their bacterial communities is rather limited. This study investigated the bacterial composition and diversity in the digestive tract (different gut sections and the infrabuccal pockets (IBPs)) of two ponerine ant species (*Odontomachus monticola* Emery and *Ectomomyrmex javanus* Mayr) distributed in northwestern China using high-throughput sequencing. We found that several dominant bacteria that exist in other predatory ants were also detected in these two ponerine ant species, including *Wolbachia*, *Mesoplasma*, and *Spiroplasma*. Bacterial communities of these two ant species were differed significantly from each other, and significant differences were also observed across their colonies, showing distinctive inter-colony characteristics. Moreover, bacterial communities between the gut sections (crops, midguts, and hindguts) of workers were highly similar within colony, but they were clearly different from those in IBPs. Further, bacterial communities in the larvae of *O. monticola* were similar to those in the IBPs of workers, but significantly different from those in gut sections. We presume that the bacterial composition and diversity in ponerine ants are related to their social behavior and feeding habits, and bacterial communities in the IBPs may play a potential role in their social life.

## 1. Introduction

Ants are one of the most abundant insect groups on the earth, occupying various niches and playing various ecological roles in many terrestrial ecosystems [1]. Ants have extensively attracted the attention of biologists not only because of their evolutionary successful social organization, but also because they have developed a fascinating behavioral repertoire. One of the most striking features of ants is that they have built close associations with other organisms in the course of exploiting food resources in nature [2], including intimate associations with bacteria to occupy diverse trophic levels [3,4].

Bacteria are considered to be a major force in driving evolution of ants, and they are one of the compelling participants in ant dietary differentiation [5]. For example, Rhizobiales are the most prevalent bacteria in most herbivorous ants, and play important roles in waste nitrogen recycling or atmospheric nitrogen fixation for related host ants (e.g., *Cephalotes* and *Tetraponera*) [6,7,8,9]. The prevalence of Rhizobiales is found to change with carbohydrate supplementation in the giant tropical ant *Paraponera clavata* (Fabricius) [10]. The intracellular *Blochmannia* co-evolves with *Camponotus* ants and contributes to nutritional upgrading and nitrogen recycling [11,12].

Bacteria can be transmitted horizontally from the environment to host insects along with food flow, and the digestive tract of insects provides ideal habitats and abundant nutrients for bacterial colonization [13]. The digestive tract of ants is characterized by a developed crop, an elaborate proventriculus, and an infrabuccal pocket (IBP) which is a special filtrating structure in the anterior pharynx, adapted to their special feeding behavior [14]. The different parts of the digestive tract can harbor disparate bacterial communities because of their different functions and physiological properties. In *Cephalotes* ants, for instance, Rhizobiales were dominant in the crop and proventriculus, but *Opitutus* was dominant in the midgut and hindgut [6]. In most ant species, however, the information on bacterial patterns in the digestive tract is still limited.

Ponerine ants are true predators, forming a primitive group in Formicidae with a simple social organization but possessing a high diversity in morphology, ecology, and behavior [15]. The gut bacterial communities in ponerine ants get less attention than herbivorous ants. Caetano et al. [16,17] found several unknown microorganisms in the midgut of *Odontomachus bauri* Emery using transmission electron microscopy (TEM), but the identity of these microorganisms are still unknown. Oliveira et al. [18] investigated the bacterial communities in the midgut of five ponerine species (i.e., *Dinoponera lucida* Emery, *Neoponera curvinodis* (Forel), *Pachycondyla striata* Smith, *Odontomachus brunneus* (Patton), and *O. bauri* Emery in Brazil), and revealed that several bacterial groups were predominant in these ants with obvious interspecific difference. Previous studies provided basic information regarding bacteria just in the midgut of ponerine ants, but the comprehensive descriptions of bacterial composition and diversity in more species and, particularly in their different gut sections, are scarce; moreover, factors affecting gut bacterial communities in ponerine ants are still unknown.

In China, the richness of ponerine ant species decreases from the south to the north, and their distributions are sensitive to change with environmental conditions [19]. To date, only four ponerine ant species have been documented in the Shaanxi Province, which is located in northwestern China, including the Qinling Mountains, the border of the subtropical and temperate zones, as well as the Parlearctic and Oriental Regions [20,21,22]. Among them, *Odontomachus monticola* Emery and *Ectomomyrmex javanus* Mayr are two widely distributed ponerine species that nest in soil, litter, or rotted wood and prey particularly on small arthropods. Further, their body size is big enough to dissect the digestive tract to compare the features of bacterial communities in different gut sections [15,19].

In the present study, we investigated the bacterial composition and diversity in the digestive tract of these two ponerine ant species using high-throughput sequencing of the bacterial 16S rRNA gene, aiming to reveal bacterial communities between species, colonies, different gut sections (crops, midguts, and hindguts), and the IBPs. Our results will provide more valuable information for the characteristics of gut bacterial communities in predatory ants, deepening our understanding of the relationship between microorganisms and the feeding habits and gut structures of ants.

## 2. Materials and Methods

### 2.1. Ant Collection and Dissection

The ants were collected in Shaanxi Province, China, from June to August 2017. All samples and their numbers are listed in Table 1. Live colonies were collected and brought to the laboratory as soon as possible, and they were stored in plastic cases with access to sterilized water but no food supply, and dissected within 48 h. Three colonies were collected for *O. monticola*, workers and larvae were included in colonies O1 and O2, but only workers were included in colony O3 at the time of collection. Two colonies were collected for *E. javanus* and only foraging workers were included; their real nest was not found.

For *O. monticola* and *E. javanus*, the IBP was dissected from the head of the surface sterilized worker as performed by Zhang et al. [23]. The whole gut was carefully exposed from the abdomen, and the crop, midgut, and hindgut were carefully separated and stored in different sterilized centrifuge tubes [24]. The IBP of workers in colony O3 of *O. monticola* could not be obtained during dissection as it was empty and undetectable. Ten of the above individual gut parts and the IBPs were merged as a pooled sample. For larvae of *O. monticola*, each complete individual was surface sterilized with 70% ethanol for 2 min, rinsed with sterile ddH_2_O, and finally transferred into 1.5 mL sterile centrifuge tubes with five individuals merged as a pooled sample. To minimize the risk of cross-contamination, dissections were performed on sterile glass slides; forceps were flame sterilized between dissections, and all procedures were conducted on a clean bench. All samples were kept in sterile centrifuge tubes at −80 °C until DNA extraction. In total, 63 samples were obtained, including *O. monticola* (*n* = 39) and *E. javanus* (*n* = 24) (Table 1). During sample preparation, we used sterile ddH_2_O and ddH_2_O rinsed off from surface sterilized ants, each gut sections, and the IBPs as negative controls, and a total of 12 negative control samples were used for subsequent genomic DNA extraction and PCR amplification [25].

### 2.2. Genomic DNA Extraction

Each pooled sample was homogenized using sterile pestles and incubated at 37 °C overnight in the lysozyme to lyse the cell walls of Gram-positive bacteria [26]. Genomic DNA was extracted using DNeasy Blood & Tissue extraction kit (Tian Gen, Beijing, China) according to the manufacturer’s instructions. All samples were finally eluted in 50 μL of TE buffer. The concentration and purity of the extracted DNA was measured using a Nanodrop 1000 Microvolume Spectrophotometers (Thermo Fisher Scientific, Waltham, MA, USA). Qualified samples were kept at −80 °C until sequencing.

### 2.3. DNA Library Construction, PCR and Illumina Hiseq Sequencing

Sequences of the V3–V4 regions of the 16S rRNA genes were amplified using primers 338F (5′-ACTCCTACGGGAGGCAGCA-3′) and 806R (5′-GGACTACHVGGGTWTCTAAT-3′), combined with adapter sequences and barcode sequences. The PCR was conducted in a 50 μL mixture containing 10 μL buffer, 0.2 μL Q5 High-Fidelity DNA polymerase (TaKaRa, Dalian, China), 10 μL High GC Enhancer, 1 μL dNTP mixture, 10 μM of each primer, and 60 ng genome DNA. Thermal cycling conditions were: an initial denaturation at 95 °C for 5 min, followed by 15 cycles at 95 °C for 1 min, 50 °C for 1 min, and 72 °C for 1 min; and a final extension at 72 °C for 7 min. The amplified PCR products from the first step were detected using 1.8% agarose gel electrophoresis, and samples with a bright main strip were chosen as they were considered in good quality, and they were purified through Vahtstm DNA Clean Beads. A second round PCR was then performed in a 40 μL reaction system that contained 20 μL 2× Phμsion HF MM, 8 μL ddH_2_O, 10 μM of each primer, and 10 μL PCR products from the first step. Thermal cycling conditions were: an initial denaturation at 98 °C for 30 s, followed by 10 cycles at 98 °C for 10 s, 65 °C for 30 s, and 72 °C for 30 s, with a final extension at 72 °C for 5 min. Finally, all PCR products were quantified by Quant-iT™ dsDNA HS Reagent and pooled together.

Finally, high-throughput sequencing of bacterial 16S rRNA gene was performed on the purified and pooled samples using the Illumina Hiseq 2500 platform at Biomarker Technologies Corporation (Beijing, China). Although no PCR products were detected in negative control samples, we also run the negative control samples with high-throughput sequencing to detect contaminants that may be present in low abundance, or other possible contaminants derived from the environment and process of DNA extraction and sequencing.

### 2.4. Bioinformatic Analysis

After sequencing, raw reads were filtered using Trimmomatic v0.33 [27], and the forward and reverse primer sequences were identified and removed with Cutadapt 1.9.1 to acquire high quality reads, and the overlapping regions between the high quality reads were merged into clean reads using Flash v1.2.7 [28]. Then, reads were denoised and chimeras were removed with Data2 [29] in Qiime2 [30], and they were conducted on feature classification to output an amplicon sequence variants (ASVs) table. Then, ASVs were clustered at 99% sequence identity, and assigned into taxonomic groups based on the SILVA database with naive Bayes classifier [31]. Negative controls were filtered using the Decontam package based on the frequency method in R v4.0.3 following default settings [32]. After feature table was filtered, ASVs classified as chloroplasts, mitochondria, and archaeal were deleted in Qiime2 and only bacterial ASVs were retained. With respect to methods used in bioinformatic analysis of high-throughput sequencing data, 97% similarity was traditionally used as gold standard to cluster sequences into OUTs. However, in recent years 99% similarity is proposed to be better than 97% similarity to deal with the sequences of microbial communities, and ASV methods are explicitly intend to replace OTUs as the atomic unit in bioinformatic analysis [33]. Therefore, in this study we applied 99% similarity and ASV methods to analyze all sequences.

In order to evaluate the bacterial composition and abundance between different groups, all samples were divided into: (a) different species, (b) different colonies, and(c) different gut sections and larvae. Alpha and beta-diversity analyses were performed with the q2-diversity plugin in Qiime2. Alpha diversity was evaluated by the Chao1 and Shannon indices to estimate the bacterial richness and diversity. Principal coordinate analysis (PCoA) was performed by using Qiime2 software based on the Bray–Curtis distance to evaluate the bacterial community composition. Statistical differences between groups were conducted with PERMANOVA and ANOSIM analyses using Vegan in Qiime2 based on the Bray–Curtis distance with 999 permutations. Biomarker discovery with linear discriminant analysis effect size (LEfSe) analysis based on non-parametric Kruskal–Wallis rank sum test was conducted in MicrobiomeAnalyst (https://www.microbiomeanalyst.ca/MicrobiomeAnalyst/home.xhtml) to explore bacterial genera that differed significantly in relative abundance between different groups, followed by discriminant analysis to evaluate the effect size of those significant genera [34]. The *p*-values were adjusted with the FDR method, and Log LDA score = 2 was the cutoff value. Bacterial genera with adjusted *p*-values < 0.05 were regarded having significantly different in abundance between the compared groups.

The raw sequences of the V3–V4 regions of the 16S rRNA genes generated in this study have been submitted to the NCBI Sequence Read Archive (SRA) databases under accession number PRJNA512110.

## 3. Results

### 3.1. Characteristics of Sequence Data

In total, 3,956,904 raw reads were produced, 3,789,308 clean reads were obtained, and 3,614,755 non-chimeric reads were applied to further analysis after merging, filtering, and chimera removal with Data 2. Each sample contained 57,377 (mean value) non-chimeric reads. The length of the reads per sample ranged from 350 to 440 bp. After assembling, these reads were clustered into 556 unique ASVs.

The rarefaction curves reached the plateau for all samples (Figure S1), suggesting that the sequencing amount of this study was sufficient for samples to cover all bacterial communities.

### 3.2. Bacterial Communities at the Phylum Level

**Figure 1:**
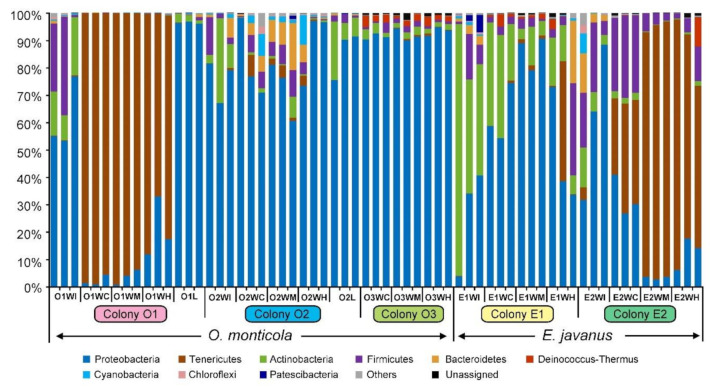
Bacterial communities of two ponerine ant species and their colonies at the phylum level. O1, O2, and O3, three colonies of *Odontomachus monticola*; E1 and E2, two colonies of *Ectomomyrmex javanus*. WI, the infrabuccal pockets of workers; WC, the crop of workers; WM, the midguts of workers; WH, the hindguts of workers; L, larvae. Sample names are listed in Table 1.

At the phylum level, nine phyla were detected in all samples, including Proteobacteria, Tenericutes, Actinobacteria, Firmicutes, Bacteroidetes, Deinococcus-Thermus, Cyanobacteria, Chloroflexi, and Patescibacteria (Figure 1).

For the gut sections of *O. monticola*, the composition and abundance of the dominant bacterial phyla varied among the three colonies but had a high similarity among different gut sections (i.e., crops, midguts, and hindguts) within each colony (Figure 1). In colony O1, Tenericutes (O1WC, 97.70 ± 1.88%; O1WM, 96.28 ± 2.68%; O1WH, 78.83 ± 10.07%) was the predominant group, followed by Proteobacteria. In colony O2, the dominant group was Proteobacteria (O2WC, 81.89 ± 14.36%; O2WM, 72.68 ± 10.72%; O2WH, 89.02 ± 13.57%), followed by Cyanobacteria, Firmicutes, Bacteroidetes, and Tenericutes. In colony O3, Proteobacteria (O3WC, 90.90 ± 1.57%; O3WM, 91.15 ± 1.12%; O3WH, 90.59 ± 2.34%) was predominant. The IBPs of workers in colonies O1 and O2 have similar dominant bacterial phyla: Proteobacteria (O1WI, 61.73 ± 13.09%; O2WI, 75.89 ± 7.71%), Actinobacteria (O1WI, 15.47 ± 6.08%; O2WI, 14.24 ± 14.76%), and Firmicutes (O1WI, 20.94 ± 17.96%; O2WI, 5.93 ± 6.76%). The dominant bacterial groups of larvae in colonies O1 and O2 were similar to those in the IBPs, including Proteobacteria (O1L, 96.38 ± 0.37%; O2L, 85.68 ± 8.86%) and Actinobacteria (O1L, 2.31 ± 1.00%; O2L, 11.84 ± 8.58%).

For the gut sections of *E. javanus*, the dominant bacterial phyla varied a lot between two colonies (E1 and E2). In colony E1, Proteobacteria (E1WC, 59.89 ± 7.23%; E1WM, 82.03 ± 5.08%; E1WH, 47.58 ± 21.51%) and Actinobacteria (E1WC, 31.12 ± 10.17%; E1WM, 7.46 ± 5.62; E1WH, 12.33 ± 5.21%) were dominant in all gut section, whereas Tenericutes (14.19 ± 23.99%), Firmicutes (13.21 ± 17.65%), and Bacteroidetes (7.60 ± 13.16%) were abundant in hindguts as well. In colony E2, Tenericutes (E2WC, 29.65 ± 4.95%; E2WM, 74.93 ± 20.96%; E2WH, 65.35 ± 5.59%), Proteobacteria (E2WC, 27.52 ± 6.89%; E2WM, 2.61 ± 0.65%; E2WH, 11.61 ± 6.27), and Firmicutes (E2WC, 23.81 ± 0.76%; E2WM, 3.47 ± 0.72%; E2WH, 6.27 ± 5.7%) were dominant. For the IBPs of workers, Actinobacteria (E1WI, 58.08 ± 29.43%; E2WI, 14.59 ± 8.37%), Proteobacteria (E1WI, 26.19 ± 19.62%; E2WI, 61.40 ± 28.42%), and Firmicutes (E1WI, 8.24 ± 7.97%; E2WI, 16.80 ± 10.51%) were the dominant groups in both colonies E1 and E2.

### 3.3. Bacterial Communities at the Genus Level

**Figure 2:**
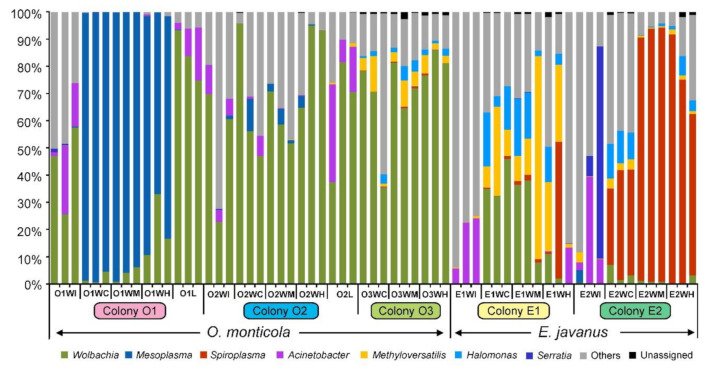
Bacterial communities of two ponerine ant species and their colonies at the genus level. O1, O2, and O3, three colonies of *O. monticola*; E1 and E2, two colonies of *E. javanus*. WI, the infrabuccal pockets of workers; WC, the crop of workers; WM, the midguts of workers; WH, the hindguts of workers; L, larvae. Sample names are listed in Table 1.

At the genus level, bacterial composition and abundance showed apparent differences among the two ponerine ant species and their colonies, and some dominant genera were found, mainly including *Mesoplasma*, *Wolbachia*, and *Spiroplasma* (Figure 2).

For the gut sections of *O. monticola*, the dominant bacterial genus varied between three colonies. In colony O1, *Mesoplasma* (O1WC, 97.70 ± 1.88%; O1WM, 96.28 ± 2.68%; O1WH, 78.83 ± 10.78%) was predominant, followed by *Wolbachia*. In colony O2, *Wolbachia* (O2WC, 59.96 ± 31.41%; O2WM, 50.46 ± 5.61%; O2WH, 81.72 ± 21.67%) was dominant, followed by *Mesoplasma*. In colony O3, *Wolbachia* (O3WC, 61.19 ± 22.55%; O3WM, 71.98 ± 7.39%; O3WH, 78.16 ± 2.69%) was predominant, followed by *Methyloversatilis* and *Halomonas*. The dominant bacteria in the IBPs were similar between colonies O1 and O2, including *Wolbachia* (O1WI, 43.36 ± 16.25%; O2WI, 45.73 ± 23.53%) and *Acinetobacter* (O1WI, 14.11 ± 12.00%; O2WI, 10.62 ± 6.56%). For larval samples from colonies O1 and O2, their dominant groups were *Wolbachia* (O1L, 84.04 ± 9.26%; O2L, 63.20 ± 22.97%) and *Acinetobacter* (O1L, 10.71 ± 8.49%; O2L, 20.32 ± 14.20%).

For gut sections of *E. javanus*, the bacterial communities of the two colonies (E1 and E2) showed distinct differences. In colony E1, the dominant bacterial groups in the crops and midguts were *Wolbachia* (E1WC, 25.32 ± 7.40%; E1WM, 24.76 ± 15.67%), *Methyloversatilis* (E1WC, 10.83 ± 8.46%; E1WM, 27.79 ± 30.59%), and *Halomonas* (E1WC, 8.84 ± 6.64; E1WM, 12.18 ± 9.43%), but dominant bacteria in the hindguts were *Spiroplasma* (14.15 ± 24.03%), *Methyloversatilis* (15.41 ± 12.21%), *Halomonas* (4.74 ± 5.51%), and *Acnitobacter* (4.27 ± 7.20%) as well. In colony E2, *Spiroplasma* (E2WC, 29.65 ± 4.95%; E2WM, 74.93 ± 20.96%; E2WH, 65.35 ± 5.59%) was dominant in all gut sections, followed by *Halomonas*. For the IBPs, the dominant genus in colony E1 was *Acinetobacter* (14.36 ± 11.99%), and dominant genera in colony E2 were *Serratia* (28.44 ± 42.90%), and *Acinetobacter* (17.03 ± 19.57%).

### 3.4. The Diversity and Similarity Analyses of Bacterial Communities

The Chao1 and Shannon indices were used to estimate the richness and diversity of the bacterial communities in all samples. Chao 1 and Shannon indices in *E. javanus* were both higher than *O. monticola* (Table S1). Chao 1 index and Shannon index in the IBPs were higher than gut sections (crops, midguts, and hindguts) within colonies O1, E1, and E2, except in colony O2 (Table S2).

**Figure 3:**
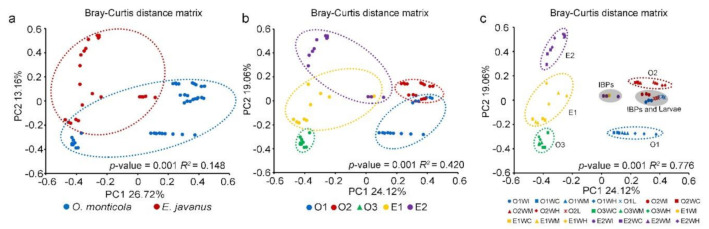
Principal coordinate analysis (PCoA) of bacterial community composition and abundance among different groups. (**a**) Bray–Curtis distance matrix for samples from *O. monticola* and *E. javanus*. (**b**) Bray–Curtis distance matrix for samples from different colonies of each ant species. (**c**) Bray–Curtis distance matrix for samples from infrabuccal pockets, gut sections (crops, midguts, and hindguts) of workers and larvae of *O. monticola* and *E. javanus*. Dots in different color represented different groups. PERMANOVA tests showed statistical analysis between groups. O1, O2, and O3, three colonies of *O. monticola*; E1 and E2, two colonies of *E. javanus*. WI, the infrabuccal pockets of workers; WC, the crop of workers; WM, the midguts of workers; WH, the hindguts of workers; L, larvae. Sample names are listed in Table 1.

PCoA analysis indicated a clear separation of bacterial communities between the two ant species (Figure 3a). Most samples from the same colony were grouped together and differ from other colonies (Figure 3b). For samples within each colony, gut samples (crops, midguts, and hindguts) of workers were obviously clustered together, forming the colony-specific groups with similar bacterial communities. Especially, six samples of the IBPs in colonies E1 and E2 formed a special group, and their bacterial communities were dissimilar with respective gut sections. In addition, samples of the IBPs and larvae in colonies O1 and O2 formed another group, showing more similar bacterial communities (Figure 3c). PERMANOVA tests revealed that bacterial communities between groups were significantly different (*R*^2^ > 0, *p* = 0.001).

**Figure 4:**
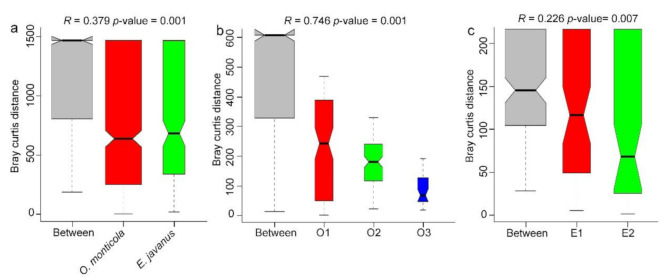
ANOSIM analysis presenting the similarity of bacterial communities among different groups. (**a**) Similarities of bacterial communities between *O. monticola* and *E. javanus*. (**b**) Similarities of bacterial communities across three colonies (O1, O2, and O3) of *O. monticola*. (**c**) Similarities of bacterial communities between two colonies (E1 and E2) of *E. javanus*.

ANOSIM results showed that there were significant differences in the bacterial communities between the two ant species (*R* > 0, *p* = 0.001) (Figure 4a), and significant differences were also found in the bacterial communities between three colonies of *O. monticola* (*R* > 0, *p* = 0.001) (Figure 4b) and two colonies of *E. javanus* (*R* > 0, *p* = 0.007) (Figure 4c). It is noticeable that bacterial communities in the IBPs of workers differed significantly from those in gut sections (*R* > 0, *p* < 0.05, respectively), but they showed no significant difference compared with those in larvae of the same colony (*R* > 0, *p* > 0.05) (Table S3).

### 3.5. Differences in the Relative Abundance of Bacterial Genera

**Figure 5:**
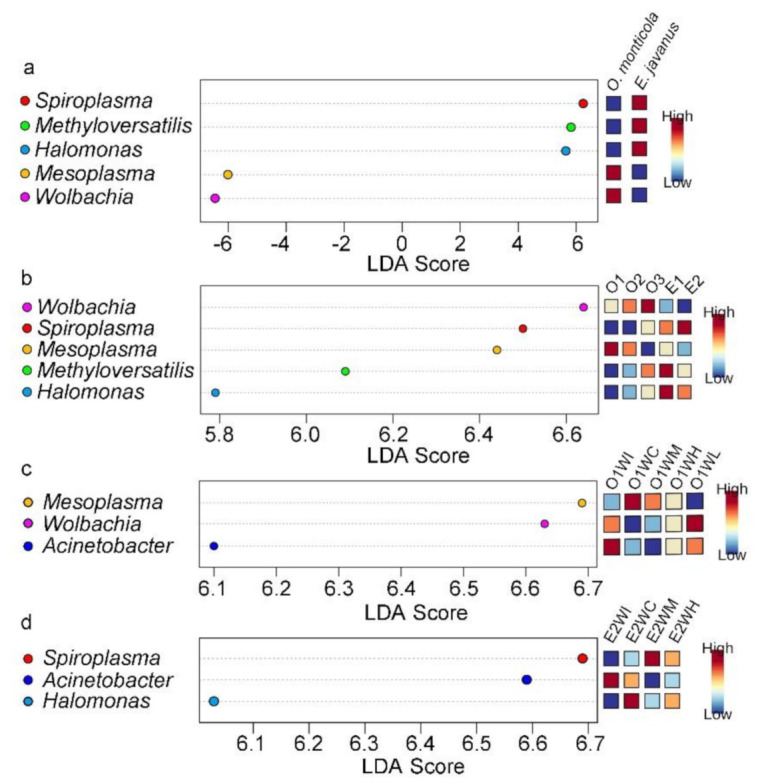
Bacterial genera that significantly differ (FDR adjusted *p*-value < 0.05) in abundance among different groups. (**a**) Bacterial genera that significantly differ between *O. monticola* and *E. javanus*. (**b**) Bacterial genera that significantly differ across three colonies (O1, O2, and O3) of *O. monticola* and two colonies (E1 and E2) of *E. javanus*. (**c**) Bacterial genus that significantly differ between samples within colony O1. (**d**) Bacterial genera that significantly differ between samples within colony E2. WI, the infrabuccal pockets of workers; WC, the crop of workers; WM, the midguts of workers; WH, the hindguts of workers; L, larvae. Significant bacterial genera were ranked in decreasing order based on their LDA score (*x* axis). The mini heatmap to the right of the plot indicated whether the relative abundance of bacterial genera were higher (red) or lower (blue) in each group.

*Wolbachia* and *Mesoplasma* were bacterial genera that had a significantly high relative abundance in *O. monticola*, but *Spiroplasma*, *Halomonas*, and *Methyloversatilis* were significantly abundant in *E. javanus* (Figure 5a). Across colonies of *O. monticola* and *E. javanus*, *Mesoplasma*, *Wolbachia*, and *Spiroplasma* were significantly abundant in colonies O1, O3, and E2, respectively, whereas *Methyloversatilis* and *Halomonas* were significantly abundant in colony E1 (Figure 5b). Within colony O1, *Mesoplasma*, *Wolbachia*, and *Acinetobacter* were significantly high in the crops (O1WC), larvae (O1L), and IBPs (O1WI), respectively (Figure 5c). In colony E2, *Spiroplasma*, *Acinetobacter*, and *Halomonas* were significantly high in the midguts (E2WM), IBPs (E2WI), and crops (E2WC), respectively (Figure 5d). The bacterial genera in different samples within colonies O2, O3, and E1 were not significantly different.

## 4. Discussion

Symbiotic bacteria are regarded as a driving force in facilitating the convergent evolution of predatory to herbivory in ants [3,5]. Ponerine ants are a primitive and predatory group in Formicidae, occupying a high trophic level (δ^15^N_predator_-δ^15^N_plant_ ≥ 3.99), and they usually harbor different microbiota from herbivorous ants which occupy a lower trophic level (δ^15^N_herbivore_-δ^15^N_plant_ ≤ 3.76) [5,35,36]. In this study, we investigated the bacterial communities in gut sections (crops, midguts, and hindguts) and the IBPs of workers in two ponerine ants *O. monticola* and *E. javanus* using Illumina Hiseq high-throughput sequencing. Our results showed that the bacterial communities and dominant groups of the two ponerine ants were clearly different between species. Furthermore, similar findings have reported in several ant species, including *D. lucida*, *N. curvinodis*, *P. striata*, *O. brunneus*, *O. bauri* [18], *Solenopsis invicta* Buren, *S. geminata* (Fabricius) [37], *Colobopsis riehlii* Roger, *Camponotus floridanus* (Buckley), and Ca. *planatus* Roger [38]. Tropic level, host phylogeny, and diet are main factors that can affect the bacterial communities between ant species [3]. With respect to this study, *O. monticola* and *E. javanus* are both predators that occupy a similar trophic level. However, *O. monticola* were distributed in undisturbed mountain areas, nesting in soil or rotting wood, and foraging for some arthropods [19]; *E. javanus* were distributed in a plain humid area, nesting in humus-rich soil, and mainly preying on small arthropods carcasses [39]. Thus, host phylogeny, diet, and nesting environment are probably main factors leading to differences of bacterial communities between two ant species.

In addition to displaying inter-species differences, bacterial communities also showed apparent inter-colony characteristics in this study. This has been shown in previous studies involving colonies of *Cephalotes varians* (Smith), *Eciton burchellii* (Westwood), *Daceton armigerum* (Latreille), *Atta sexdens rubropilosa* Forel, *Pseudomyrmex flavicornis* (Smith), and *P. nigrocinctus* (Emery) [25,40,41,42,43]. The differences in bacterial communities among colonies might be due to the genetic variability of the ant hosts [40,42]. In addition, environmental acquisition and geographic isolation possibly play roles in differing bacterial communities between colonies; therefore, after long-term adaption to the nest environment, ants possibly form colony-specific gut bacterial communities.

We further compared the bacterial communities in different parts of the digestive tract of these two ponerine ants for the first time. The results showed that the bacterial communities were highly similar between each gut sections (crops, midguts, and hindguts) of workers, but significantly different from the IBPs within each colony. This phenomenon may be related with the trophallaxis behavior and the gut structures in different ant groups. Trophallaxis is a special behavior involving exchange of food between nestmates in advanced ants, in which foraging workers bring food back to the nest and transfer liquid to another, but it was believed to be absent in primitive ponerine ants [1,44]. However, previous studies showed that some ponerine ants rely on pseudotrophallaxis to share food between nestmates by surface tension in the mandible without real regurgitation, examples were found in *Pachycondyla* (*=* *Neoponera*) *villosa* (Fabricius) [45] and *O. troglodytes* Santschi [46]. Moreover, the structure of the proventriculus of ponerine ants is not as elaborate as that of formicine and dolichoderine ants [44], and it cannot dam the efflux of the crop contents effectively. Thus, it allows bacteria to move backwards with food flow to the midgut and hindgut, leading to the similarity of bacterial communities between gut sections.

The IBP is a special pouch-like filtration device located in the preoral cavity of ants [47]. The anterior part of the IBP and the inner wall of the prepharynx, which are covered with long hairs, form an effective filtering system for solid materials [48,49]. In the fungus-growing ants, the IBP can prevent the invasion and spread of the specific fungus-garden parasite *Escovopsis* in their fungus garden [50]. We have previously isolated diverse bacterial strains in the IBPs of Ca. *japonicus* Mayr, and the dominant genera were *Acinetobacter* and *Microbacterium* [51]. Subsequently, by means of high-throughput sequencing, we found that the bacterial abundance and diversity in the infrabuccal pockets were higher than those in the crops and midguts [23]. In this study, we found that bacterial communities of the IBPs from four colonies (O1, O2, E1, and E2) of two ponerine ant species indeed contain certain dominant bacteria, however their bacterial communities were significantly different from gut sections (Table S3, *p* < 0.01). Meanwhile, Chao 1 and Shannon indices of bacterial communities in the IBPs were also higher than gut sections within colonies O1, E1, and E2, except for colony O2 (Table S2). Thus, the IBP may obstruct the passage of certain bacteria to the gut, leading to the differences of bacterial communities in the IBPs compared to the guts. However, we further found that bacterial communities in the IBPs of *O. monticola* were similar to those in larvae. This is the first time to provide a very interesting hint about the relationship between the IBPs and larvae in terms of bacterial communities. It was reported that ponerine ants *Platythyrea* exhibited larva-to-worker trophallaxis [52], and workers of Pseudomyrmecinae ants have been found to place the food pellets formed in the IBP into the trophothylax of the larvae as a food source [53]; the same behavior was also found in the fire ant *S. invicta* Buren [54]. Therefore, we think that the similarity of bacterial communities between the IBPs and larvae provides an indirect evidence that larva-to-worker trophallaxis exists in *O. monticola* and *E. javanus*. Further studies are needed to confirm the trophallaxis between larvae and adults, and the potential functions of the IBP in the social life of ponerine ants.

Previous studies revealed that the trophic level has a stronger impact in shaping bacterial communities than a specialized diet [55]; we found that the bacterial communities of two ponerine species were obviously different from those of the herbivorous ants [6,7,8,9,10,11,12]. Although bacterial communities between the two ant species presented differences, several dominant bacterial groups were identified among them, including *Wolbachia*, *Spiroplasma*, and *Mesoplasma*. These bacteria have been documented in the midgut of five ponerine ant species with 16S rRNA RFLP methods [18], such as *Spiroplasma* (90.7% clones) in *D. lucida*, *Wolbachia* (46.1% clones) and *Mesoplasma* (52.6% clones) in *N. curvinodis*, *Spiroplasma* (99.4% clones) in *P. striata*, *Spiroplasma* (68.0% clones) and *Mesoplasma* (32.0% clones) in *O. brunneus*, and *Serratia* (87.1% clones) in *O. bauri*. *Mesoplasma* and *Spiroplasma* which affiliated with Entomoplasmatales are host-specific across species of three army ant subfamilies (Aenictinae, Dorylinae, and Ecitoninae) [56]. Combined with these previous studies, our results indicated that ponerine ants might harbor host-specific bacterial groups, including Entomoplasmatales (*Mesoplasma* and *Spiroplasma*) and *Wolbachia*.

*Wolbachia* is commonly found in the germline of insects as an intracellular symbiont, and it was believed to be mainly transmitted vertically from mother to offspring through eggs. However, more studies found that *Wolbachia* was not only presented in the reproductive tissues of insects but also observed in somatic tissues, including the nervous system, fat body, gut, salivary glands, malpighian tubules [57,58,59,60,61]. Here, we found that *Wolbachia* nearly occurred in all samples across colonies of *O. monticola* and *E. javanus*, in gut sections (crops, midguts, and hindguts), and in the larvae and the IBPs in the head. We had not expected such a widespread distribution of *Wolbachia* in ants, but some studies on *Wolbachia* showed that it can be found in the heads of several species of *Drosophila*, mosquitos, tsetse fly, and termites [61]; thus *Wolbachia* may have a wide distribution in the somatic tissues of ants. Previous studies showed that nearly 35% of ants are infected with *Wolbachia*, for example *Acromyrmex*, *Formica*, *Solenopsis*, *Camponotus*, *Cephalotes*, and *Tetraponera* [62,63,64,65]. Although it was documented to influence the sex ratio in *Monomorium pharaonis* (Linnaeus) [66], we still know little about its functions within ants. *Mesoplasma* has been observed in attine ants [43,67,68,69], and the genomic information of two *Mesoplasma* strains revealed that they play important roles in decomposing arginine and providing nitrogen-rich amino acid [70]. In this study, *Mesoplasma* was found to be predominant in colony O1 of *O. monticola*, accounting for 97.23 ± 2.06% of the bacterial communities in the crop, 95.82 ± 2.61% in the midgut, and 77.29 ± 10.08% in the hindgut; thus, it might be acquired horizontally from the environment, or its presence may be related to the physical condition of the nest. *Spiroplasma* was only found in colony E2 of *E. javanus*, and it has been detected in the ant genera *Polyrhachis*, *Cephalotes*, *Pseudomyrmex*, and *Tetraponera* [71]; further, it is commonly considered unnecessary for host development and reproduction [56]. Hence, the functions of these dominant bacteria in ants still need to be elucidated.

## 5. Conclusions

The two ponerine ants investigated in this study harbor dominant bacterial groups (*Mesoplasma*, *Spiroplasma*, and *Wolbachia*) similar to those in other predator ants, and those bacterial groups possibly play a certain role in the social behavior and feeding habits of ponerine ants. The relationship of the bacterial communities among the infrabuccal pockets, gut sections and larvae provide meaningful information that can reveal the potential functions of these microbes in the ecology and social evolution of ants.

## Figures and Tables

**Table 1:** Information of 63 samples from the two ponerine ants in this study.

Ant Species	Colony IDs	Samples	Collection Sites and Nesting Habits	GPS Location
*Odontomachus monticola* Emery	O1	O1WI (infrabuccal pockets), O1WC (crops); O1WM (midguts), O1WH (hindguts), O1L (larvae)	Fengxiang County, nesting in soil	34°54′60.4″ N 107°53′36.8″ E
	O2	O2WI (infrabuccal pockets), O2WC (crops), O2WM (midguts), O2WH (hindguts), O2L (larvae)	Linyou County, nesting in soil	34°68′35.3″ N 107°79′52.6″ E
	O3	O3WC (crops), O3WM (midguts), O3WH (hindguts)	Ningshan County, nesting in rotting wood	33°40′36.4″ N 108°38′08.1″ E
*Ectomomyrmex javanus* Mayr	E1	E1WI (infrabuccal pockets), E1WC (crops), E1WM (midguts), E1WH (hindguts)	Yangling County, nesting in soil	34°28′86.4″ N 108°07′77.4″ E
	E2	E2WI (infrabuccal pockets), E2WC (crops), E2WM (midguts), E2WH (hindguts)		34°26′38.2″ N 108°07′83.2″ E

## Data Availability

The data that support the findings of this study are available from the first and corresponding authors [Z.Z. and H.H.] upon reasonable request.

## Supplementary Materials

The following are available online at https://zenodo.org/record/4480390#.YBTEsnGf480, Figure S1: Rarefaction curves of bacterial ASVs for all samples, Table S1: Diversity indices of bacterial communities between the two ponerine ant species, Table S2: Diversity indices of bacterial communities in the infrabuccal pockets and guts of workers and larvae, Table S3: ANOSIM analysis of bacterial communities across infrabuccal pockets and guts of workers, and larvae: *p*-values.
